# Non-invasive brain stimulation for osteoarthritis

**DOI:** 10.3389/fnagi.2022.987732

**Published:** 2022-09-29

**Authors:** Hui-Qi Zhu, Jing Luo, Xue-Qiang Wang, Xin-An Zhang

**Affiliations:** ^1^College of Kinesiology, Shenyang Sport University, Shenyang, China; ^2^Department of Sport Rehabilitation, Xi’an University of Sport, Xi’an, China; ^3^Department of Sport Rehabilitation, Shanghai University of Sport, Shanghai, China; ^4^Department of Rehabilitation Medicine, Shanghai Shangti Orthopaedic Hospital, Shanghai, China

**Keywords:** osteoarthritis, NIBS, rTMS, single-pulse TMS, tDCS, tRNS, tACS, tFUS

## Abstract

Osteoarthritis (OA) is a degenerative joint disease, the prevalence of OA is increasing, and the elderly are the most common in patients with OA. OA has a severe impact on the daily life of patients, this increases the demand for treatment of OA. In recent years, the application of non-invasive brain stimulation (NIBS) has attracted extensive attention. It has been confirmed that NIBS plays an important role in regulating cortical excitability and oscillatory rhythm in specific brain regions. In this review, we summarized the therapeutic effects and mechanisms of different NIBS techniques in OA, clarified the potential of NIBS as a treatment choice for OA, and provided prospects for further research in the future.

## Introduction

As a general degenerative disease and the most common form of arthritis, osteoarthritis (OA) is pathologically characterized by cartilage degeneration, subchondral bone sclerosis, and osteophyte formation (Pereira et al., 2015). Cartilage degeneration is also a hallmark of OA (Egloff et al., 2012). The major clinical symptoms of OA are joint pain and activity disorders. The new subchondral bone tissue that results from subchondral bone sclerosis and osteophyte formation contains new blood vessels and nerve fibers, which may be related to OA pathogenesis and pain perception (Walsh et al., 2010). These in turn cause the patient’s dysfunction, poor sleep, and low mood; these conditions seriously affect the quality of life (QOL) of patients. In accordance with statistics, the prevalence rate of OA in people over 60 years old can reach 50%, it in people over 75 years old is as high as 80%, the prevalence in women is higher than that in men, and the disability rate of the disease can reach 53% (Zhang et al., 2020). Patients with OA worldwide are expected to exhibit an increasing trend in the next few decades (Mantovani et al., 2016) partly due to increasing risk factors for OA, such as genetics, obesity, lack of exercise, or exercise injury (Hawker, 2019; Kakouris et al., 2021). The disease not only exerts a huge influence on the QOL of patients but also considerably increases the economic burden of society. At present, however, the complex pathological mechanism of OA is not yet fully known; it mostly involves increased inflammatory components, mechanical overload, altered metabolic changes, and cellular aging (Jeon et al., 2017). In contrast with other inflammatory joint diseases, varying responses to different parts of OA further complicate treatment, and thus, treatment for OA remains a challenge (Hermann et al., 2018).

OA is currently mainly treated with drugs (Guo et al., 2022), mainly non-steroidal anti-infective drugs, which have anti-inflammatory and analgesic effects, but these drugs have many adverse reactions, and long-term use can lead to different degrees of cardiovascular complications (da Costa et al., 2017). Intra-articular injections such as glucocorticoids can reduce joint adhesion and promote cartilage repair, but they only have short-term effects, and long-term injections may cause infection (Kavanaugh et al., 2016). Cartilage protective drugs such as glucosamine can reduce joint edema, maintain synovial fluid viscosity, and inhibit inflammation, but long-term use has serious adverse reactions to gastrointestinal function and is expensive (Arden et al., 2021). Joint-targeted drug therapy only works in the diseased part and has no effect on other normal parts, which is conducive to cartilage repair and pain relief (Zhang et al., 2022). However, the current targeted therapy is still in the clinical trial stage, and the potential adverse reactions are not yet clear. Therefore, it is considered to explore a non-drug-safe treatment method for OA.

OA is also mostly treated with physical therapy, mainly exercise therapy, hydrotherapy, and extracorporeal shock wave therapy (Griffin et al., 2020). Anti-blocking exercise combined with hydrotherapy is prone to adverse events such as worsening pain (Waller et al., 2017) and other symptoms in patients with moderate to severe OA, and hydrotherapy has had only a small short-term clinical effect in patients with OA (Bartels et al., 2016) and does not appear to have a significant effect on the patient’s drug dose or quality of life (Forestier et al., 2016). Extracorporeal shock wave therapy (ESWT) is important for the protection of articular cartilage in patients with early OA, but it can only slow down the course of OA. Moreover, the experimental subjects are mostly mice, and the doses for humans have not been standardized (Chou et al., 2019). Whole-body vibration therapy also does not improve joint stiffness in patients with OA (Qiu et al., 2022).

Bioelectronic medicine uses the body’s electrical signals to improve the diagnosis and treatment of disease. As an emerging bioelectronic medical technology, Non-invasive brain stimulation (NIBS) has been widely used in clinical treatment, particularly for pain (Xiong et al., 2022), neuronal function regulation, brain function cognition, behavior, and other aspects of the evident treatment effect (Miniussi and Ruzzoli, 2013). NIBS not only brings hope for treating diseases that cannot be solved by common drugs and medical means, but also provides drug alternatives with rapid and precise targeting. This paper describes the therapeutic effects and possible mechanisms of six commonly used NIBS methods in OA, namely single pulse transcranial magnetic stimulation (TMS), repetitive transcranial magnetic stimulation (rTMS), transcranial direct current stimulation (tDCS), transcranial alternating current stimulation (tACS), transcranial random noise stimulation (tRNS), transcranial focused ultrasound stimulation (tFUS), to provide directions for future research.

## Methods

A comprehensive literature search was conducted in PubMed and Web of Science databases using [Osteoarthritis(Title/Abstract)] OR [OA(Title/Abstract)], [Osteoarthritides(Title/Abstract)] OR [Osteoarthrosis(Title/Abstract)] OR [Osteoarthroses(Title/Abstract)] OR [Arthritis, Degenerative(Title/Abstract)] OR [Arthritides, Degenerative(Title/Abstract)] OR [Degenerative Arthritides(Title/Abstract)] OR [Degenerative Arthritis(Title/Abstract)] OR [Arthrosis(Title/Abstract)] OR [Arthroses(Title/Abstract)] OR [Osteoarthrosis Deformans(Title/Abstract)], [Non-invasive brain stimulation(Title/Abstract)] OR [NIBS(Title/Abstract)], rTMS[Title/Abstract] and Osteoarthritis, Single-pulse TMS[Title/Abstract] and Osteoarthritis, tDCS[Title/Abstract] and Osteoarthritis, tFUS[Title/Abstract] and Osteoarthritis, tACS[Title/Abstract] and Osteoarthritis, tRNS[Title/Abstract] and Osteoarthritis. From the date initially provided until November 2021, it is not limited to randomized controlled trials (RCT). Firstly, irrelevant articles were excluded according to the article titles or abstracts, and after reading and summarizing the remaining articles, the articles with high relevance, in-depth research, or lack of research were selected for inclusion.

## Non-invasive brain stimulation

**FIGURE 1:**
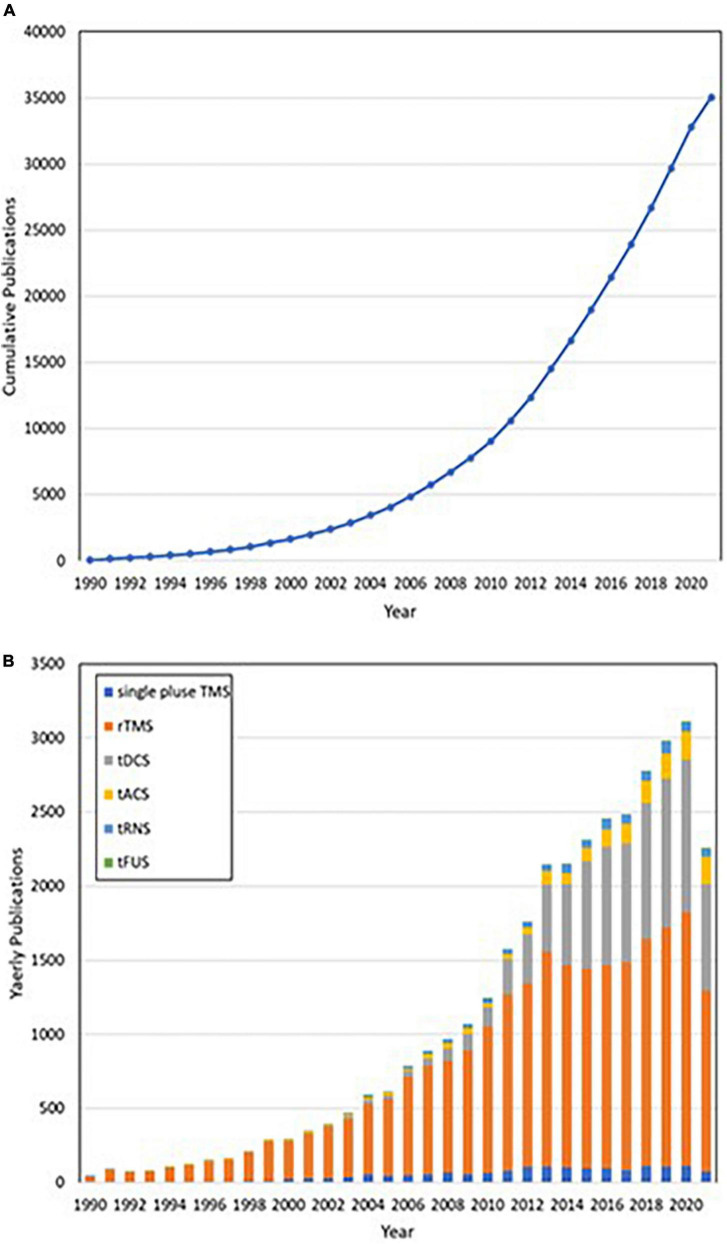
**(A)** Publications accumulated by year. **(B)** From 1990 to 2020, publications were classified annually in accordance with different non-invasive brain stimuli in categories that included repetitive transcranial magnetic stimulation (rTMS, orange), single-pulse transcranial magnetic stimulation (single-pulse TMS, blue), transcranial direct current stimulation (tDCS, gray), transcranial alternating current stimulation (tACS, yellow), transcranial random noise stimulation (tRNS, light blue), and transcranial focused ultrasound stimulation (tFUS, green).

NIBS has become an effective and multi-field treatment technology in recent years. It is used to study the behavioral correlation of specific brain regions; neuronal regulatory function and its related perceptual, cognitive, and behavioral characteristics play a huge role (Plewnia et al., 2015), significantly deepening researchers’ understanding of NIBS. However, NIBS is more dependent on brain state and task content, and some variability can be observed even among or within individuals. And the long-term effects of NIBS have not been explored (Polanía et al., 2018). Recent research trends on NIBS are presented in Figures 1A,B.

Clinically, common NIBS technologies include TMS (Ansari et al., 2021) and transcranial electrical stimulation (tES) (Abhishek and Doherty, 2013; Terranova et al., 2018). Among TMS techniques, single-pulse and rTMS are more commonly used. Meanwhile, tES can be divided into tACS and tDCS. In addition, tFUS (Badran et al., 2020) and tRNS (Battaglini et al., 2020) are also commonly used methods of NIBS (O’Connell et al., 2018).

TMS is a late-model neurophysiological technique based on the theory of the principle of electromagnetic induction (Barker et al., 1985; Hallett, 2007). Its principle is to use the rapidly changing magnetic field that emerges when a high-voltage current passes through a coil, acting on a certain region of the cerebral cortex, or the corresponding skull surface; hence, the magnetic field produces an induction current, which, in turn, changes the neural cell excitability in that region (Klomjai et al., 2015). Such changes can be recorded on effectors through the neural pathway. TMS is divided into three categories: single pulse TMS, double pulse TMS, and rTMS (Auvichayapat and Auvichayapat, 2009; Ansari et al., 2021). There is also an emerging TMS model called Theta Burst Stimulation (TBS). rTMS is a new neuroelectrophysiological technology. rTMS not only affects the local function of the cerebral cortex, but relatively distant cortical function can also achieve cortical function reconstruction. High-frequency TMS can enhance the metabolism of nerve cells, while low-frequency TMS can inhibit nerve cell metabolism (Du et al., 2019). Thus rTMS can produce lasting changes in cortical excitability (Pell et al., 2011) and has become a new treatment for neurological disorders. However, rTMS remains in the preliminary exploration stage. Its treatment effect and optimal treatment parameters have not been scientifically confirmed, and its treatment mechanism also requires further research and discussion (D’Agati et al., 2010). Single-pulse TMS emits only one stimulation pulse at a time; such pulse can depolarize neurons and cause a measurable response (Mathew and Danion, 2018), primarily for the detection of neural pathways, i.e., motion-evoked potential or measurement of a motor threshold. Single-pulse magnetic stimulation can also be used to detect central conduction time, and resting time, accurately map the motor cortex, and measure the motor evoked potential amplitude related to motor cortical conduction (Zorn et al., 2012). However, the aforementioned studies, have no targeted disease or standardized parameters. Dual-pulse magnetic stimulation refers to the emission of two consecutive pulses or one pulse pair after each issued instruction; it can be used in the research on cortical excitability (de Goede et al., 2020). TBS is similar to the frequency of theta waves in the hippocampus of the brain and is a special type of rTMS. It uses short pulses in clumps, with an intra-frequency of 50 Hz and an inter-frequency of 5 Hz (Ferrarelli and Phillips, 2021), which has the advantages of short stimulation time, long duration of therapeutic effect, and closer to the physiological state of neurophysiological activity. Different TBS models have different effects on neuronal excitability. Intermittent TBS (iTBS) acts by continuous stimulation for 2 s with 8 s intervals, which can increase neuronal excitability. On the other hand, continuous TBS (cTBS) provides continuous stimulation at a frequency of 5 Hz, which reduces neuronal excitability (Bai et al., 2022). This article mainly describes two ways of treating OA, rTMS and Single-pulse TMS.

tDCS (Ahn et al., 2019a) is a pair of electrodes that use constant, low-intensity current (1–2 mA) on specific brain regions to regulate cortical neural activity (Cucca et al., 2019). It is widely used in the clinical treatment of Parkinsonism and other neurological diseases; it can also be used to improve motor, language, cognitive, and swallowing functions (Santos Ferreira et al., 2019). However, the exact mechanism of tDCS is not yet completely clear. Its clinical application remains in the incremental research phase, and a single mechanism cannot explain the multiple effects of tDCS; moreover, no unified scheme is available for the choice of stimulation intensity, time, and location of tDCS (Palm et al., 2016a). Currently, for tDCS, the widely recognized mechanism of action is the effect on membrane potential and ion channels, synaptic plasticity, cortical excitability (Nitsche and Paulus, 2000), bilateral hemispheric excitability, and regulation of local cortical and brain network connections (Lapenta et al., 2018).

tACS (Antal et al., 2008) is a special mode of NIBS that transmits sinusoidal alternating current electricity to the scalp, mostly affecting the excitability of cortical neurons (Bland and Sale, 2019). The currently recognized action mechanism of tACS is to regulate brain function by guiding brain oscillations (Antal et al., 2008) and inducing synaptic plasticity over a long period to regulate cognitive processes (Elyamany et al., 2021); it can also link cellular neuronal activity to brain network mechanisms to recover disturbed brain oscillations and perfect behavioral outcomes (Del Felice et al., 2019). tACS can be used as a practical means to judge the diagnosis, classification, and prognosis of mental diseases. tACS is relatively safe and non-invasive (Matsumoto and Ugawa, 2017); hence, it exhibits considerable potential in basic research and as a tool for clinical care. However, information on how tACS ultimately affects neural activity (Vieira et al., 2020) is minimal, and the two practical factors of tACS and electric field are difficult to focus on accurately, which is a common problem in tACS; thus, this technique should be further optimized and studied (Wu et al., 2021).

tRNS is a type of TMS, which exhibits the potential to induce improvements with a lasting perception when combined with assignments such as a contrast detection test (Terney et al., 2008). Nevertheless, the mechanism of these long-range improvements is not yet fully determined. tRNS can strengthen contrast sensitivity after one training cycle; however, this early onset also depends on the characteristics of the stimulus (Battaglini et al., 2020). tRNS can sense the effect of duration (Mioni et al., 2018). Interference with persistent neuronal oscillations can ultimately generate neuroplasticity effects if suitable parameters are applied (Paulus, 2011).

tFUS has a higher spatial resolution and can reach deeper organizations compared with other NIBS methods (di Biase et al., 2019). tFUS acting on the brain can modulate human cortical function (Legon et al., 2014). For example, two 10-min tFUS in the front of the thalamus will induce analgesic effects in healthy individuals (Badran et al., 2020). tFUS can also be used to regulate emotion regulatory networks in the prefrontal cortex (Goh et al., 2020; Sanguinetti et al., 2020). Low-intensity tFUS enhances the neuromodulatory effect of human autonomous motor-related cortical activity (Yu et al., 2021). Through an in-depth study, tFUS was found to be safe, and adverse reactions rarely occur. However, under high stimulation intensity or rate, tFUS may lead to bleeding, cell death or injury, and accidental opening of the blood–brain barrier. Thus, the study of tFUS is necessary to set up a good and secure framework for the application and promotion of clinical treatment of tFUS (Pasquinelli et al., 2019).

**FIGURE 2:**
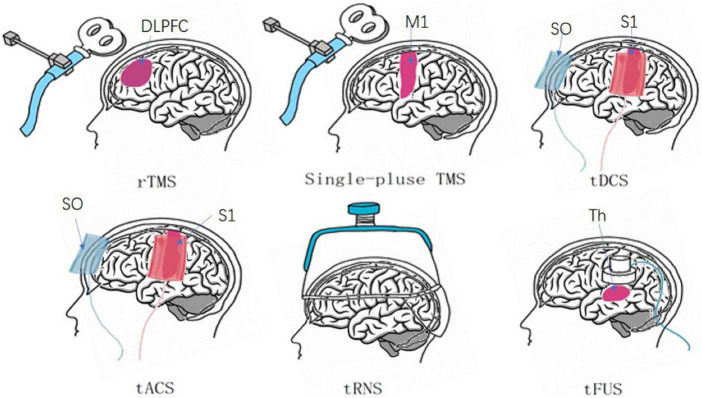
Six major non-invasive brain stimuli and their site of action. rTMS generally functions in the dorsolateral PFC (DLPFC) (Lefaucheur et al., 2020). Single-pulse TMS uses frequent sites of action, such as the primary motor cortex (M1) (Savoie et al., 2020). tDCS and tACS cathode and anode positions: contralateral orbit (SO), primary sensory cortex (S1) (Marlow et al., 2013; May et al., 2021). tRNS: cerebral cortex. tFUS: thalamus (Th) (Palm et al., 2016b; Badran et al., 2020).

The six methods of NIBS are commonly used in clinical treatment, and they exhibit unique characteristics, including their respective sites of action, as indicated in Figure 2.

## Therapeutic effects of non-invasive brain stimulation on osteoarthritis

### Relieve pain

Pain is a common symptom in patients with OA (Perrot, 2015), and with an increase in incidence in OA and the risk factors that affect OA, pain places heavy burdens on the daily life of patients with OA and society. The treatment of pain of patients with OA is particularly important. NIBS has been shown to exhibit a therapeutic function on the pain brought by OA in many studies. For example, Jean-Paul Nguyen et al. (2019) performed a monthly rTMS treatment of 10 Hz in the right motor cortex of an elderly female patient suffering from left knee OA. One week after the third treatment, the pain was significantly reduced, and after 1 year of follow-up, the effect of pain reduction continued. Ahn et al. (2017) performed a tDCS intervention of 2 mA per day in middle-aged and elderly patients with knee OA, placing the anode on the primary motor cortex (M1) and the cathode on the contralateral supraorbital (SO). After five interventions, the patient’s NRS scores decreased significantly and the Short-Form McGill Pain Questionnaire (SF-MPQ-2) scores also improved. The therapeutic effect of pain relief was found to be maintained after the 3-week follow-up. Ahn et al. (2018) also evaluated the pain sensitivity of 400 elderly patients with knee OA and conducted five tDCS interventions. Their results showed that the experimental pain sensitivity of the patients was reduced, and tDCS exerted a beneficial effect on the improvement of clinical pain. tFUS is frequently used in chronic neuropathic pain. However, targeted studies on the pain of OA are few. Research on tACS, Trns, and single-pulse TMS with regard to the pain of OA remains in its infancy and requires further exploration.

### Alleviate symptoms of depression

Depression and other bad moods are closely related to OA. The occurrence of OA may aggravate depressive symptoms (Zheng et al., 2021), and the long-term accumulation of these symptoms may aggravate the pain grade and joint dysfunction of patients with OA (Rathbun et al., 2018, 2020). NIBS has been widely used in research related to depressive symptoms. Depression assessment, such as the Hamilton Depression Rating Scale, was used as a secondary evaluation index in the research of Nguyen et al. (2019) on the program of rTMS intervention in the treatment of OA. Patients with 20 years of OA showed a significant increase in their HAD scores after ten rTMS interventions. This suggests that rTMS is effective in treating depression in patients with OA. Ahn et al. (2019b) evaluated the efficacy of remotely supervised tDCS for the treatment of OA patients, elderly KOA patients did not experience significant improvement in anxiety and depression scores after 10 consecutive tDCS interventions. This may be the result of various factors, such as autonomously improper use of tDCS. Luz-Santos et al. (2017) and Tavares et al. (2018) also studied tDCS for depression in OA patients, although the results were not available. This requires further research by researchers. The other basic NIBS methods have minimal research on the depressive symptoms of OA, and more studies are still required to confirm their result.

### Improve joint motor function

The articular cartilage, as an important basic structure of joints, is the major structure affected by OA, which can cause joint deformities, joint swelling, and a decrease in the range of motion (Abhishek and Doherty, 2013), resulting in limited motor function in patients with OA. In severe cases, joint replacement surgery should be considered. Drug therapy is mostly used in the early treatment of OA, but the efficacy of drug therapy is limited (Cao et al., 2022). The safety and non-invasive characteristics of NIBS make it indispensable in the auxiliary treatment of OA. In a randomized controlled trial of tDCS combined with conventional physical therapy on the functional ability of patients with OA, Fatemeh Rahimi et al. (2021) used the anode of tDCS to intervene in the left primary motor cortex (Ahn et al., 2017), primary sensory cortex (Ahn et al., 2018), and dorsolateral PFC. The subjects were evaluated functionally with the Keen Injury and OA Outcome Score rating scale, the range of motion of the knee joint, 30-s chair standing, and 4 × 10-m walking training before and after treatment. The results show that various functional indicators were significantly improved after tDCS intervention. The strength of the muscles around the joints is also an important factor affecting joint function. Quadriceps weakness is a typical symptom of KOA. Kittelson et al. (2014) performed TMS stimulation on subjects with this symptom. After stimulation, quadriceps torque was reduced, resting motion threshold was also decreased, and the Western Ontario and McMaster Universities OA Index (Luz-Santos et al., 2017) scores increased. All these findings suggested the improvement of the motor function of subjects. The effects of tACS, tRNS and other methods on joint motor function have not yet been confirmed.

### Quality of life

As a common degenerative disease of the elderly, OA is becoming increasingly in many young individuals at present, and it is one of the major factors that affect the daily activities and QOL of patients (Sabashi et al., 2021). Studies have shown that (Paget et al., 2021) the QOL of young people is more affected by OA than that of older patients in ankle OA. In the research protocol of Tavares et al. (2018) on elderly KOA patients with defective endogenous pain-inhibitory systems, health-related QOL (HRQOL) was included in the evaluation indicators of OA subjects after tDCS intervention. In the subsequent trial (Tavares et al., 2021), 2 mA tDCS intervention at the stimulation site of M1 and SO for 20 min at 15 times in 3 weeks did not significantly improve the HRQOL and WOMAC scores, and did not exert an evident effect on the QOL of patients with OA. However, in patients with OA after total knee replacement surgery, the SF-36 score was significantly improved, and the QOL of the patients was also improved after 6 weeks of treatment with electroacupuncture and exercise therapy combined with tDCS (Li et al., 2021). The curative effect of NIBS in the QOL of OA patients remains uncertain, and other factors should be excluded for further research. The detailed process and results of other research are provided in Table 1.

**TABLE 1:** Characteristics of the research literature of the six NIBS methods for the treatment of OA.

Authors/ Publication time	Journal	*N*	Stimulation				Evaluation indicators	Experimental results
			Treatment methods	Duration	Intensity	Rate		
**rTMS**								
Ansari et al. (2021) 2021-4	ACS Chem Neurosci	_____________						rTMS can improve muscle fibrous pain.
Nguyen et al. (2019) 2019-4	Front Neurosci	1 (71 years old, female, KOA)	rTMS	10 months	10 Hz	Once a month	HAD, LISO, NRS	rTMS is particularly appealing to treat pain associated with KOA
**Single-pulse TMS**								
_____________								
**tDCS**								
Sajadi et al. (2020) 2020-10	Neurophysiol Clin	40 (Adult, KOA)	Knee strengthening exercises + tDCS, knee strengthening exercises + TENS	2 weeks	1–2 mA	Six times	VAS?WOMAC	Effects of tDCS and TENS were not significantly different on the pain and function of patients with KOA
Fillingim et al. (2020) 2020-11	Contemp Clin Trials	60 (NHBs and NHWs with KOA)	Four groups of BAT (real vs. sham) + tDCS (real vs. sham)	1 week	1–2 mA	Five times a week	QST, WOMAC, SPPB	–
Pollonini et al. (2020b) 2020-11	J Neuroimaging	19 (The elderly with KOA)	MBM + tDCS, sham MBM + tDCS	2 weeks	2 mA	Five times a week	fNIRS, NRS, WOMAC	Combining tDCS and MBM reduced experimentally induced pain and pain perception on KOA.
Ahn et al. (2017) 2017-9	Brain Stimul	40 (50–70-year-old, KOA)	tDCS, sham tDCS	5 days	2 mA	Once a day	NRS, WOMAC, SF-MPQ-2, 6 MWT, SPPB	tDCS was efficacious in reducing of clinical pain perception in patients with KOA.
Ahn et al. (2019a) 2019-8	J Clin Neurosci	20 (50–85-year-old, KOA)	tDCS	10 Days	2 mA	Once a day	PROMIS, VAS, WOMAC, SF-MPQ	tDCS was feasible and beneficial in alleviating pain in older adults with KOA
Pollonini et al. (2020a) 2020-4	Neurophotonics	10 (9 females, 1 male, 62.4 ± 6.9 years, OA-related pain suffering 37.7 ± 31.5months, affected by right KOA from the greater Houston community)	tDCS	2 weeks	2 mA	Five times a week	VAS, WOMAC, fNIRS	tDCS can increase cortical excitability and alleviate pain in patients with KOA.
Teixeira et al. (2020) 2020-4	Princ Pract Clin Res	Adult, KOA > 3 months	tDCS + PTM, PTM alone, PTM + sham tDCS	_____________			VAS	Potential analgesic effect of tDCS in combination with PTM for fibromyalgia and KOA.
Ahn et al. (2018) 2018-9	J Pain Res	40 (50–70 years with KOA pain)	tDCS, sham tDCS	5 days	2 mA	Once a day	NRS, WOMAC, QST	tDCS can reduce experimental pain sensitivity and facilitate pain inhibition in older patients with KOA.
Ahn et al. (2019b) 2019-12	J Clin Neurosci	30 (50–85 years old with symptomatic KOA)	MBM + tDCS, sham MBM + sham tDCS	10 days	2 mA	Once a day	NRS, WOMAC, QST	Promising clinical efficacy of home-based tDCS paired with MBM for older adults with KOA
Rahimi et al. (2021) 2021-9	Neurophysiol Clin	80 (KOA)	PT + tDCS in M1, PT + tDCS in S1, PT + tDCS in DLPFC, sham PT + sham tDCS	1 month	2 mA	Once every 3 days	VAS, KOOS	tDCS can be a beneficial additional treatment for pain relief, disability reduction, and functional improvement in patients with KOA.
Luz-Santos et al. (2017) 2017-12	Trials	80 (KOA)	Active anodal tDCS + sham PES, sham tDCS + active PES, sham tDCS + PES, active tDCS + PES	5 days	2 mA	Once a day	SF-36, VAS, WOMAC, HAD	–
da Graca-Tarragó et al. (2019) 2019-1	J Pain Res	60 (women with KOA, aged 50–75 years old)	a-tDCS/a-EIMS, s-tDCS/s-EIMS, a-tDCS/s-EIMS, s-tDCS/a-EIMS	5 days	2 mA	Once a day	VAS, WOMAC, PPT	tDCS combing with EIMS can improve pain measures and descending pain inhibitory controls in KOA.
Suchting et al. (2020a) 2020-1	Biol Res Nurs	40 (50–70 years with KOA)	Sham tDCS, tDCS	5 days	2 mA	Once a day	IL-1β, IL-6, IL-10 TNF-α, CPR, cortisol, β-endorphin	Active Tdcs can reduce inflammation in patients with KOA.
Chang et al. (2015) 2015-8	BMJ Open	20 (age over 50 years, morning stiffness lasting less than 30 min, a minimum pain score of 40 on a 100 VAS, KOA)	Active tDCS + exercise, sham tDCS + exercise	8 Weeks	2 mA	Two times a week	VAS, WOMAC	–
Laste et al. (2012) 2012-8	Exp Brain Res	18 (rats)	Sham tDCS, tDCS	8 days	500 μA	Once a day	Von Frey test	tDCS induced significant, long-lasting, neuroplastic effects of OA
Suchting et al. (2020b) 2020-11	Pain Med	60 (aged 50–85 years, with self-reported unilateral or bilateral KOA pain)	tDCS	2 weeks	2 mA	Five times a week	VAS, WOMAC, QST	tDCS improved pain in older adults with KOA
Li et al. (2021) 2021-6	Med Sci Monit	80 (KOA who underwent TKA)	tDCS + electroacupuncture	6 weeks	1.5 mA	Five times a week	SF-36, VAS, FOOS, HSS	tDCS plus electroacupuncture effectively reduces pain following TKA
Chang et al. (2017) 2017-6	PLoS One	57 (age over 50 years, morning stiffness lasting less than 30 min, a minimum pain score of 40 on a 100 VAS, KOA)	Sham tDCS + exercise, active tDCS + exercise	8 weeks	1 mA	Two times a week	VAS, WOMAC	AT + EX may improve pain, function and pain mechanisms in KOA
Tavares et al. (2021) 2018-10	JMIR Res Protoc	94 (KOA, pain in the knee for a minimum of 6 months)	tDCS, sham tDCS	3 weeks	2 mA	Five times a week	NRS, BPI, PPT	–
Tavares et al. (2018) 2021-5	Brain Stimul	104 (age over 60 years with KOA pain and a dysfunctional DPIS)	tDCS, sham tDCS	15 days	2 mA	Once a day	BPI, the 12-item short form health survey questionnaire, MMSE, 0–100 VAS	–
**tACS**								
_____________								
**tRNS**								
_____________								
**tFUS**								
di Biase et al. (2021) 2021-6	Neurol Res Int	_____________					VAS	tFUS has huge research potential in the field of pain management.

NRS, numeric rating scale; BPI, the Brief Pain Inventory; MMSE, the Mini-Mental State Examination; VAS, 0–100 visual analog scale; WOMAC, the Western Ontario and McMaster Universities Osteoarthritis Index; PPT, pressure pain threshold; QST, quantitative sensory testing; TKA, total knee arthroplasty; KOOS, Knee Injury and Osteoarthritis Outcome Score; SF-36, The Short Form-36 Health Survey; HSS, the Hospital for Special Surgery; TNF-α, tumor necrosis factor-α; IL, interleukin; CRP, C-reactive protein; MBM, mindfulness-based meditation; HAD, the hospital anxiety and depression scale; LISO, The lequesne index of severity for osteoarthritis; BAT, Breathing and Attention Training; SPPB, Short Physical Performance Battery; fNIRS, functional near-infrared spectroscopy; SF-MPQ-2, Short-Form McGill Pain Questionnaire; PROMIS, the Patient-Reported Outcomes Measurement Information System; PTM, physical therapy modality; EIMS, intramuscular electrical stimulation.

## Mechanisms of non-invasive brain stimulation for osteoarthritis

### Controlling central sensitization

OA has a complicated physiological mechanism. Studies have shown that (Fingleton et al., 2015; Perrot, 2015; Vincent, 2020) nerve mediators such as the nerve growth factor (NGF) and central sensitization are closely related to the severity of OA pain. An excitatory–inhibitory mechanism has been proven in the corticospinal system that is related to the degree of pain and the disorder of pain downward control (Passard et al., 2007). rTMS can inhibit the corticospinal system’s excitability by stimulating it, modifying the central pain regulation system (Tarragó et al., 2016), and activating a large number of structures involved in pain processing bilaterally, leading to the long-term relief of chronic extensive pain. The high-frequency rTMS of the motor cortex can also restore the endoscopic suppression control associated with pain relief, and further reduce the pain of OA. tDCS interrupts mechanical pain processing, regulates high-order neuronal circuits that are altered by central sensitization, and weakens the mechanical stimulation response of spinal dorsal horn neurons (Meeker et al., 2019), controlling central sensitization and alleviating pain. Single-pulse TMS can also act on the motor cortex of the brain to increase the overall excitability of the corticospinal cord and the inner periphery of the patient (Woods et al., 2016), reducing quadriceps torque and improving motor function.

### Reducing inflammatory factors

Inflammation has been proven to play an important role in OA (Sokolove and Lepus, 2013). Compared with healthy people, OA patients have higher levels of inflammatory factors, such as the tumor necrosis factor-α (Hong et al., 2020), interleukin-1β (Cao et al., 2022), and IL-6 (Wang and He, 2018). The increase of these inflammatory factors may damage the articular cartilage, synovium, and other structures (Mathiessen and Conaghan, 2017); it can also cause the release of adipokines such as leptin, which indirectly causes OA. rTMS can act on microglia, induce the capability of synaptic plasticity, promote the secretion of the anti-inflammatory factor IL-10, and inhibit the production of pro-inflammatory factors, reducing inflammatory response (Lenz et al., 2021). Under the stimulation of cathodic tDCS, endogenous neural stem cells (NSCs) exhibit a tendency to increase (Ryu et al., 2009), inducing a strong regenerative response and reducing the inflammatory response. Neuritis has beneficial and harmful effects on the prevention of secondary tissue damage (Stoll et al., 2002), regeneration, and recovery. Therefore, the parameters of NIBS should be controlled to promote the beneficial aspects of inducing the recruitment of endogenous NSCs and minimizing inflammation as much as possible (Mabuchi et al., 2000). Promoting the production of factors is conducive to the normal growth of chondrocytes and slows down the development of OA.

### Influencing gene expression

Mitochondria are the cell organelles where genetic genes are replicated and transcribed; they also have their gene-mitochondrial (Blanco et al., 2018), and the variation of mtDNA directly affects OA phenotypes (Blanco et al., 2018). Therefore, different gene expressions exert varying effects on the OA process. Among them, rTMS can effectively affect the regulation of astrocytes related to the expression of pro-inflammatory or anti-inflammatory genes (Hong et al., 2020) and may change the cell membrane potential and cell function of astrocytes (Ruohonen and Karhu, 2012) to produce anti-inflammatory mediators for the neuroprotection effect, further reducing inflammation response (Liddelow et al., 2017). rTMS can promote the increase of Nrf2 nuclear metastasis (Tian et al., 2020), and Nrf2 can activate genes via antioxidant response, which can effectively protect cells under inflammatory conditions and can also inhibit the expression of signal channels that induce inflammation, effectively reducing the inflammatory response. In addition, tDCS can stimulate the regulation of osteopontin (Rabenstein et al., 2015, 2019). OPN can increase the survival, proliferation, migration, and neuronal differentiation of endogenous NSC (Rabenstein et al., 2015); enhance the proliferation and migration of neuronal precursors after cerebral ischemia (Denhardt et al., 2001), and play an important role in organizational balance, wound healing, immune regulation, and other aspects. These conditions can alleviate secondary damage in patients with OA, promote cartilage repair, and accelerate functional recovery in patients with OA. Although tACS, tFUS, and other methods still lack research on OA gene expression, NIBS plays a role in the pathological mechanism of OA gene expression.

### Improving cortex excitability

Studies suggest that higher cortical excitability may be a mechanism of OA and is closely linked to chronic pain and motor capacity in OA patients (Kittelson et al., 2014; Simis et al., 2021). OA patients with lower cortical inhibition had higher degrees of pain and a more obvious decrease in quadriceps motor function. As one of the NIBS methods for stimulating nerve cells in the superficial brain area (Fan et al., 2021), rTMS can regulate cortical excitability and recovery through different frequency stimulations (Barker, 1991). Among the rehabilitation studies based on the hypothesis of cross-hemisphere competition (Chen and Seitz, 2001), low-frequency rTMS may cause a short-term decline in the excitability of the unaffected hemisphere cortex (Wang et al., 2019), balance the excitability of the cerebellum, improve patients’ learning ability, and increase walking ability. By contrast, high-frequency rTMS can enhance the cortical excitability of the affected hemisphere during exercise (Goh et al., 2020), reducing competition and gradually improving the exercise capacity of patients. The improvement of specific motor functions by tDCS is also related to changes in the excitability of the motor cortex. The tDCS acting on M1 can increase the activity of the cerebral cortex, reduce the body’s response time, and better activate a patient’s motor function (Stagg et al., 2012). Therefore, NIBS might alleviate the symptoms of OA by improving cortical hyperexcitability.

### Adjusting endocrine

Obesity is an important risk factor for OA, and obesity is driven by complex endocrine mechanisms (Steidle-Kloc et al., 2018). Under the intervention of rTMS, the serum lipid level is significantly reduced (Ren et al., 2017), while the contents of thyroid-stimulating hormone and thyroxine are increased simultaneously. This finding indicates that rTMS can regulate serum lipid metabolism by changing the hypothalamic–pituitary–thyroid axis (George et al., 1996). Therefore, rTMS has the functions of regulating endocrine, improving PFC metabolic activity (Trojak et al., 2011), and regulating serum metabolic activity of lipid levels, thereby stabilizing blood lipid levels, reducing the incidence of obesity, and ultimately decreasing the effect of obesity on OA. tDCS is also extremely important in the control of obesity, but its specific mechanism remains unclear (Araujo et al., 2018), and the endocrine regulation mechanism that affects OA should be further explored.

**FIGURE 3:**
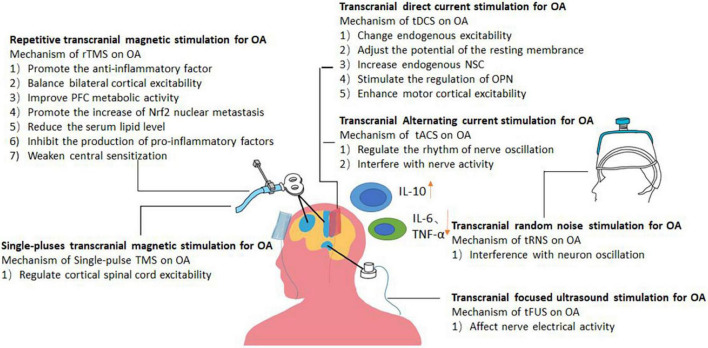
Picture showing the likely mechanism of action of different forms of NIBS in the treatment of osteoarthritis.

**FIGURE 4:**
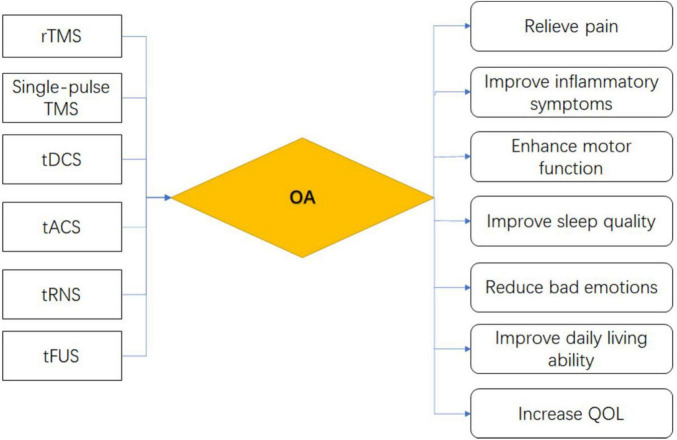
Effects of six major NIBS on OA.

In summary, on the basis of its role in complex pathophysiological mechanisms, NIBS can be further applied to OA to test its therapeutic mechanism and efficacy of OA. We summarize the mechanism and effects of six commonly used NIBS methods on OA, as shown in Figures 3, 4.

## Future perspectives

Although NIBS has been proven to be effective and feasible in the treatment of OA, there are still some limitations that need to be improved. First, existing research has focused on the tDCS and rTMS methods. Studies on OA treatment of single-pulse TMS, tACS, tRNS, and tFUS are few. Second, although NIBS, as a mature treatment technique, has been extensively applied to the treatment of clinical diseases in many fields in recent years, the treatment of OA is still in its infancy. No uniform treatment standard for regulating the treatment of OA by NIBS is available. Moreover, when OA occurs in different joints, it will exhibit different symptoms and require more targeted treatment. In addition, current NIBS studies have mostly focused on the pain symptoms of OA, and the treatment of other symptoms lacks evidence to confirm. In summary, the understanding of NIBS in the treatment of OA is limited, and more in-depth exploration can be performed from the aforementioned aspects, such that OA can be treated with more standardized NIBS, enabling OA patients to participate better in family and social life, and reduce social burdens.

## Conclusion

With the continuous research on NIBS, the feasibility and effectiveness of NIBS in the treatment of OA have been confirmed by research. This paper summarizes the current research progress of NIBS for OA, describes the main clinical effects and possible mechanisms of NIBS in treating OA, and provides a basis for further research on NIBS in the therapy of OA in the future. However, the treatment protocol of NIBS for the treatment of OA is not unified, which provides a research direction for future research.

## Author contributions

X-QW and X-AZ: draft conception, project administration, and funding acquisition. H-QZ, X-QW, X-AZ, and JL: writing – review and editing. All authors contributed to the article and approved the submitted version.
